# Supplementary material for: Alpha-1,4-transglycosylation Activity of GH57 Glycogen Branching Enzymes Is Higher in the Absence of a Flexible Loop with a Conserved Tyrosine Residue
Source: Polymers (Basel). 2023 Jun 22;15(13):2777. doi: 10.3390/polym15132777 (PMC10346327; doi:10.3390/polym15132777)
Supplement: Supplementary file 1 [file polymers-15-02777-s001.zip › polymers-2443223-supplementary.pdf]

Supplementary data

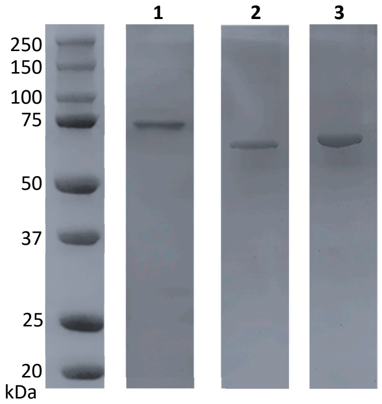

Figure S1. SDS-PAGE gel of purified TkGBE57 (*Thermococcus kodakarensis;* 79 kDa, lane 1), MsGBE57 (*Meiothermus sp., 56 kDa, lane 2*) and TmGBE57 (*Thermotoga maritima;* 63 kDa, lane 3). Protein amount on gel is 1.25 µg.

Table S1. Activity of TmGBE57 (*Thermotoga maritima)*, TkGBE57 (*Thermococcus kodakarensis)*, MsGBE57 (*Meiothermus sp.)* & EcGBE13 (*Escherichia coli*) on amylose and a linear maltodextrin (MD18), analyzed with two different methods (iodine assay and reducing end assay).

		**A**	**B**			**C**		
**Method**		Iodine assay	Reducing end assay			Reducing end assay		
**Substrate**		Amylose	Amylose			MD18		
		**Total activity [mU/mg E]**	**Non-branching activity [mU^{NB}/mg E]**	**Branching activity [mU^{B}/mg E]**	**Ratio B:NB** *	**Non-branching activity [mU^{NB}/mg E]**	**Branching activity [mU^{B}/mg E]**	**Ratio B:NB***
**Enzyme**	**TmGBE57**	84.3 ± 1.0#	2.0 ± 0.2	3.1 ± 0.3	1.5 ± 0.0	4.4 ± 1.0	5.0 ± 0.6	1.2 ± 0.2
	**TkGBE57**	440.7 ± 30.3	11.0 ± 0.5	44.9 ± 5.7	4.0 ± 0.3	13.0 ± 5.6	92.4 ± 9.5	8.1 ± 2.3
	**MsGBE57**	20.0 ± 0.7	1.0 ± 0.1	1.9 ± 0.2	2.0 ± 0.3	5.2 ± 0.3	5.6 ± 0.2	1.1 ± 0.0
	**EcGBE13**	100.5 ± 11.2	13.4 ± 8.6	133.1 ± 27.5	14.6 ± 7.3	120.1 ± 0.9	286.9 ± 42.5	2.4 ± 0.3

average of three independent measurements with standard deviation
*ratio branching activity to non-branching activity

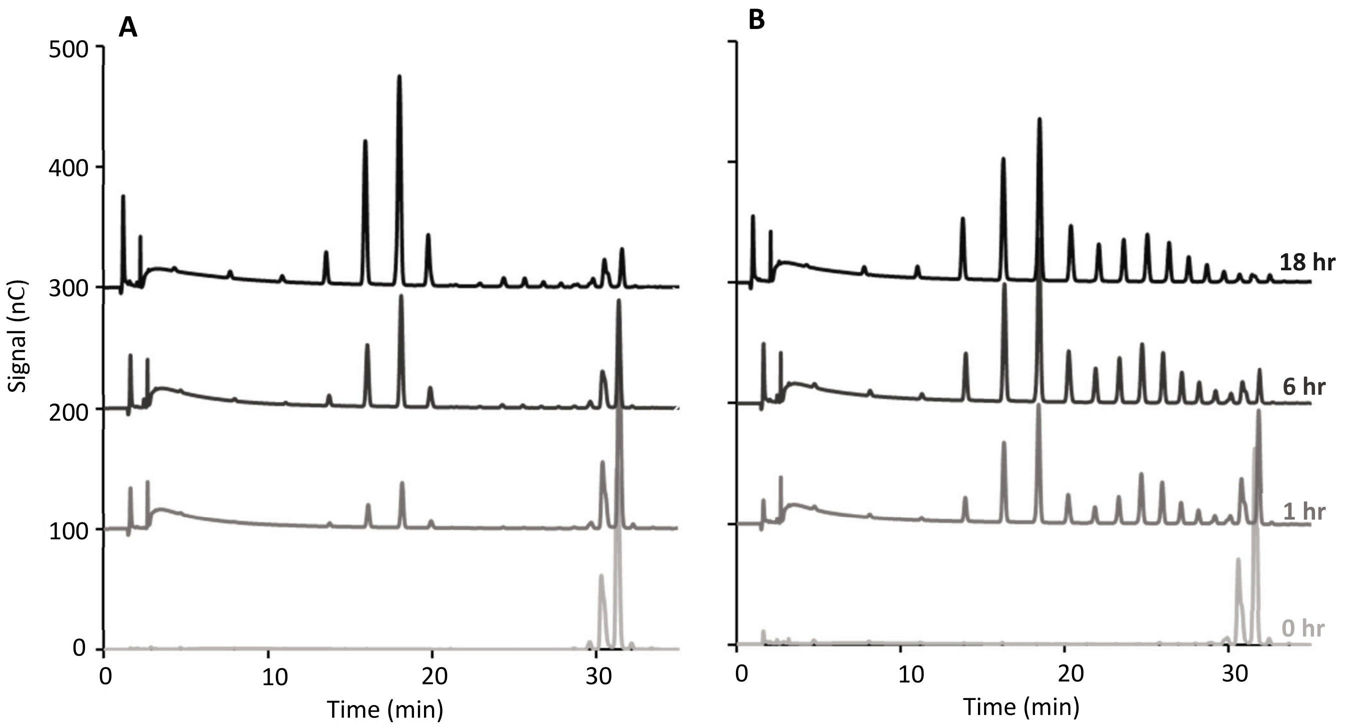

Figure S2. Chain length distribution of MD18 modified with TkGBE57 (*Thermococcus kodakarensis;* 1U^B/g S) over time before [A] and after [B] debranching compared to the untreated substrate.

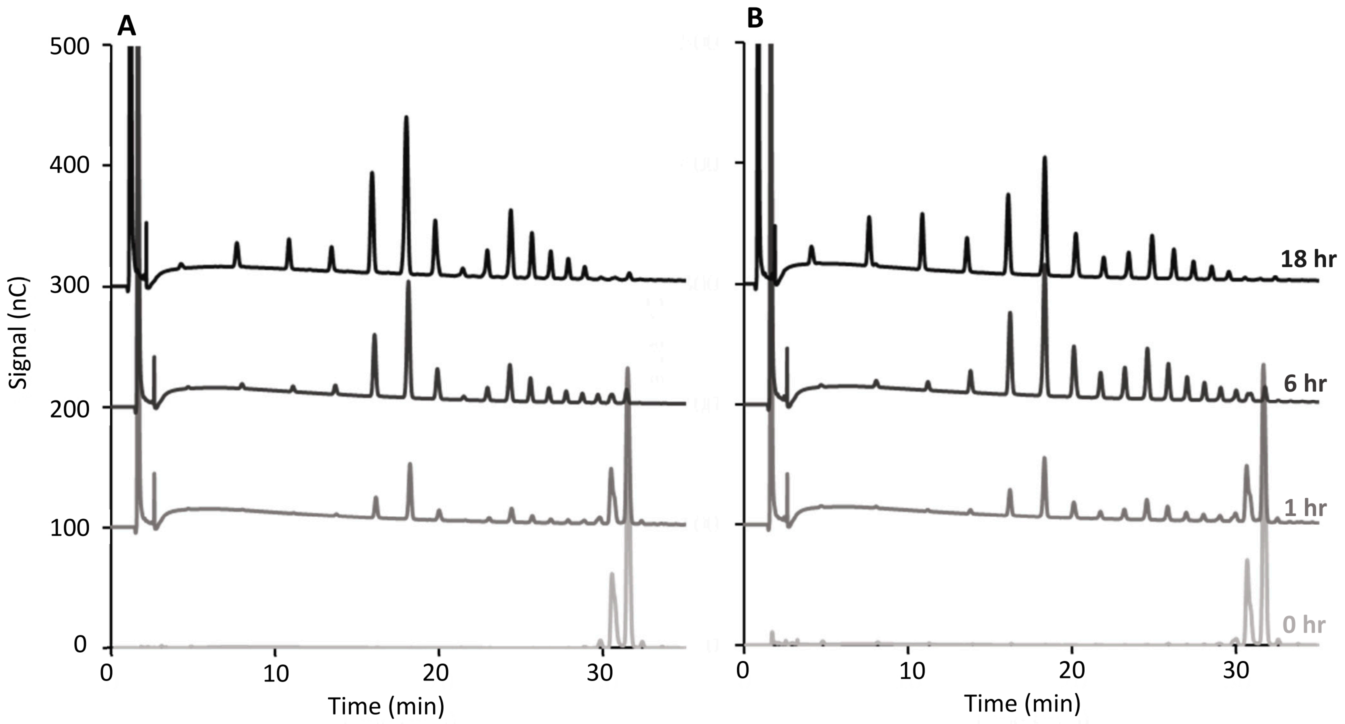

Figure S3. Chain length distribution of MD18 modified with TmGBE57 (*Thermotoga maritima;* 1U^B/g S) over time before [A] and after [B] debranching compared to the untreated substrate.

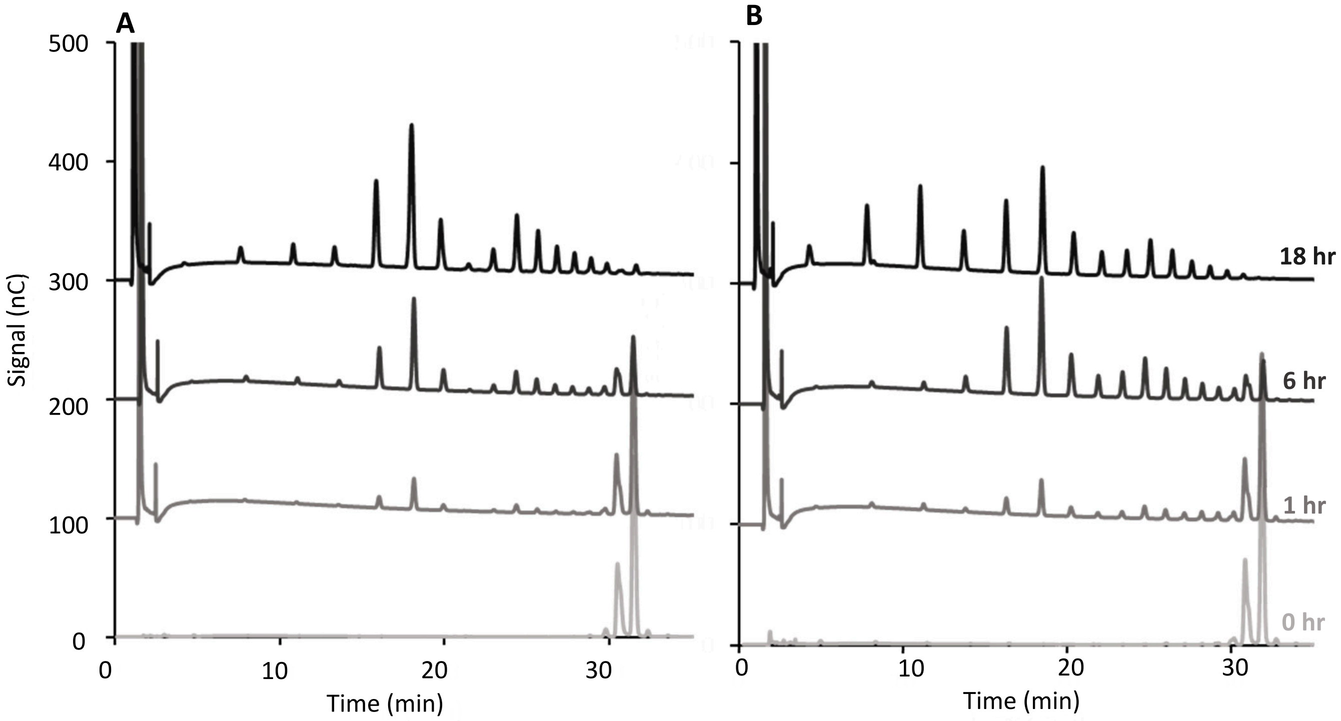

Figure S4. Chain length distribution of MD18 modified with MsGBE57 (*Meiothermus sp.;* 1U^{B}/g S) over time before [A] and after [B] debranching compared to the untreated substrate.
